# Studies on Natural Products Using Monoclonal Antibodies: A Review

**DOI:** 10.3390/antib10040043

**Published:** 2021-11-01

**Authors:** Yukihiro Shoyama

**Affiliations:** Faculty of Pharmaceutical Sciences, Nagasaki International University, 2825-7 Huis Ten Bosch, Sasebo, Nagasaki 859-3298, Japan; shoyama@niu.ac.jp

**Keywords:** monoclonal antibody, glycosides, eastern blotting, immunoaffinity separation, knockout extract

## Abstract

An immunoblotting system (“eastern blotting”) was developed for small-molecule herbal medicines like glycosides, with no conjugation function to the membrane. Briefly, the crude extracts of herb medicines were developed by thin-layer chromatography (TLC). The small-molecule herbal medicines on TLC plates were transferred to polyvinylidene fluoride (PVDF) or polyethersulfone (PES) membranes by heating. Antigen components were divided into two categories based on their function, i.e., their membrane recognizing (aglycone part) and fixing (sugar moiety) abilities. This procedure allows for the staining of only target glycosides. Double eastern blotting was developed as a further staining system for two herb medicines using a set of MAbs and substrates.

## 1. Introduction

Natural product quality is flexible, depending on plants’ cultivation and growing places, cultivation conditions, and harvest season, resulting in the development of personalized pharmacopoeias in almost all countries for the determination of natural product quality. Quality control chromatographic analyses include thin-layer chromatography (TLC), capillary electrochromatography [1], gas chromatography coupled with mass spectrometry (GC–MS) [2], high-performance liquid chromatography (HPLC), HPLC coupled with MS (LC–MS) [3], and LC–MS/MS [4] coupled with an evaporative light scattering detector [5]. A wide range of different technologies in chemistry, physics, and biochemistry contributed altogether to the rapid progress in biotechnology and molecular biosciences up to the 1970s, resulting in the production of polyclonal antibodies (PAb) against natural products. During the 1980s, monoclonal antibodies (MAb) [6] became necessary tools for immunostaining in a wide range of biological investigations. The use of several MAbs has been established in the medicinal field. However, MAbs against drugs isolated from natural products, such as morphine [7], are few.

Although the determination of hapten number in hapten-carrier protein conjugates is the most important strategy for MAb preparation, no suitable methodology is available for its confirmation. In order to confirm the hapten number, the authors of a previous study used Matrix-Assisted Laser Desorption/Ionization–TOF-Mass Spectrum (MALDI–TOF-MS) as a rapid and visual technique and succeeded in determining the molecule mass of a forskolin–bovine serum albumin (BSA) conjugate [8]. It became evident that this methodology could be applied for almost all small-molecule natural products, such as phenolics including marihuana [9], phenolic glycosides including sennoside A [10], alkaloids including codeine [11] and berberine [12], terpenoid glycosides including crocin [13], steroidal glycosides including ginsenoside Rb1 [14], glycyrrhizin [15], and solasodine glycoside [16], resulting in MAb production, as shown in Table 1.

In this review, we aim at discussing immunostaining approaches for small-molecule natural products using MAbs.

## 2. Immunostaining Using MAbs

*Glycyrrhiza* species (Leguminosae) are perennial plants native to Western Europe, Russia, China, and Mongolia. *Glycyrrhiza* root is used in more than 70% of Kampo medicines for the treatment of alimentary, respiratory, nervous, endocrine, and cardiovascular diseases [36]. The licorice root contains at least 500 compounds, including glycyrrhizin (GC), a major pharmacological active triterpene saponin-like compound used as an expectorant, anti-demulcent, anti-ulcer, anti-cancer, anti-inflammatory, or anti-diabetic agent [37].

**Figure 1:**
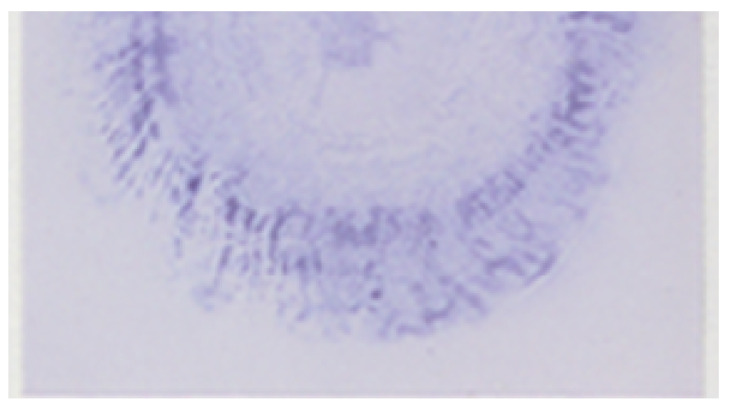
Glycyrrhizin immunocytolocalization in a fresh licorice root slice using an anti-glycyrrhizin monoclonal antibody (author’s unpublished data).

GC distribution in the licorice root tissue was confirmed by immunostaining using an anti-GC MAb [38]. The fresh root slice of licorice was covered with a polyvinylidene difluoride (PVDF) membrane and pressed. The blotted PVDF membrane was treated with a sodium periodate (NaIO4) solution followed by BSA, resulting in the formation of a GC–BSA conjugate on the PVDF membrane. The PVDF membrane was then treated with the anti-GC MAb, followed by peroxidase-labeled goat anti-IgG MAb and finally 4-chloro-1-naphthole. This method is theoretically the same as the previously described immunostaining of the TLC technique [39]. Figure 1 shows GC immunostaining in fresh licorice root, highlighting that the phloem (outer tissues) contained higher GC levels than the xylem (inner tissues).

**Figure 2:**
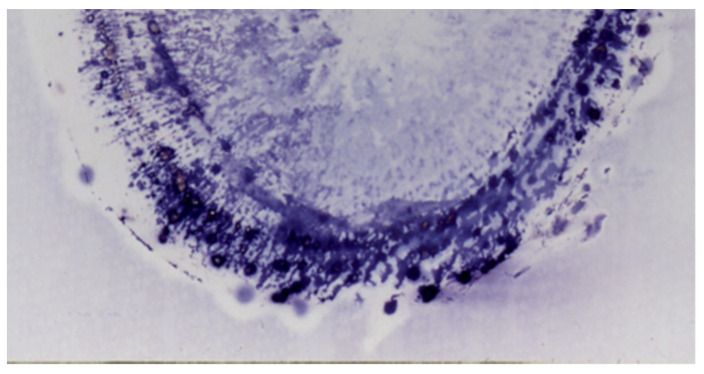
Ginsenoside Rb1 distribution in fresh ginseng root using the anti-ginsenoside Rb1 monoclonal antibody (author’s unpublished data).

Ginseng (*Panax ginseng*) is one of the oldest and most important natural products and contains several active components, including ginsenosides, triterpene saponins, polyacetylenes, polysaccharides, phenolics, alkaloids, lignans, and peptides [40]. Yang et al. [41] reported that 257 dammarane-type triterpenes, 14 octillol-type triterpenes, and 18 oleanana-type triterpenes were isolated from *Panax* species. Among them, ginsenoside, a common component of *Panax* species, promotes cholesterol and neutral lipid biosynthesis, adrenal cortex hormone secretion, and DNA and RNA synthesis and has a wide pharmacological spectrum with analgesic, antifebrile, and antifatigue properties that could alleviate sleeping troubles, improve memory and learning, and relax or excite the central nervous system [42]. A major ginsenoside, ginsenoside Rb1, could be used as a marker constituent for ginseng quality control. In the case of fresh ginseng root, almost the same results were obtained as with GC using the anti-ginsenoside Rb1 MAb, as indicated in Figure 2 [43].

**Figure 3:**
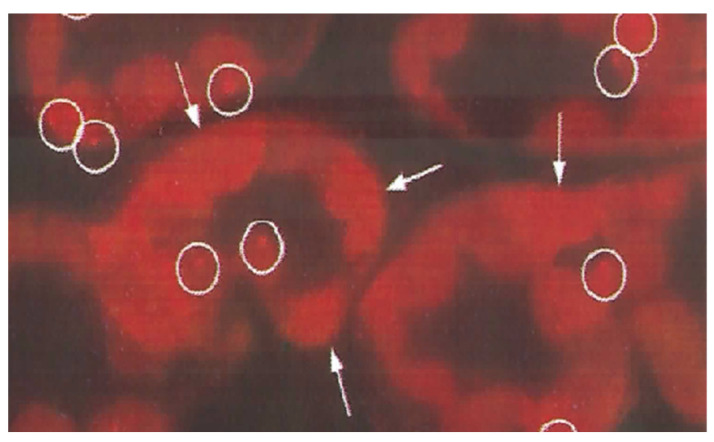
Microscopic analysis of ginsenoside Rb1 using the anti-ginsenoside Rb1 monoclonal antibody (author’s unpublished data). The circles and arrows indicate peroxisomes and chloroplasts, respectively.

A microscopic analysis of ginseng leaf tissues using the anti-ginsenoside Rb1 MAb showed more detailed information related to ginsenoside Rb1 distribution. Figure 3 indicates ginsenoside Rb1 histochemical staining in fresh ginseng leaf tissue. Peroxisomes and chloroplasts of the parenchymal cells were strongly stained [44]. These results made it clear that ginsenoside Rb1 is biosynthesized in the leaf peroxisomes and chloroplasts and is accumulated in the root, especially in the phloem tissues, as shown in Figure 2.

**Figure 4:**
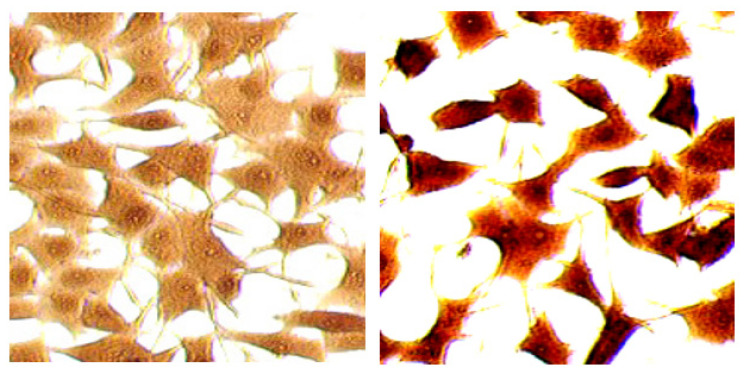
Crocin treatment in PC-12 neurons and immunostaining using an anti-crocin monoclonal antibody (author’s unpublished data). The left panel shows a non-treated control. The right panel shows staining after crocin treatment for 1 h. The black color indicates the cells stained with anti-crocin monoclonal antibodies.

We have previously reported that crocin in saffron has multifunctional activities such as strong anti-oxidant activity, improvement of learning and memory, effects on long-term potentiation, induction of non-REM sleeping, apoptosis inhibitory effect in neuronal cells, and anti-cancer activity [45]. In several investigations, the uptake of crocin into PC-12 neurons was demonstrated by immunostaining with an anti-crocin MAb [13], as shown in Figure 4.

Aristolochic acid (AA) immunostaining, associated with rapidly progressive interstitial nephritis called AA nephropathy, was investigated in mouse kidney tissues. Under light microscopy, strong immunostaining using an anti-AA MAb occurred on the apical surface of the proximal tubules [46]. The authors attempted to identify an AA target protein. After incubation of human kidney cells with AA, cell lysates were immunoprecipitated with an anti-AA MAb, followed by LC–MS analysis, which led to the identification of α-actinin-4 as an AA target protein in human kidney cells [47].

## 3. Eastern Blotting

**Figure 5:**
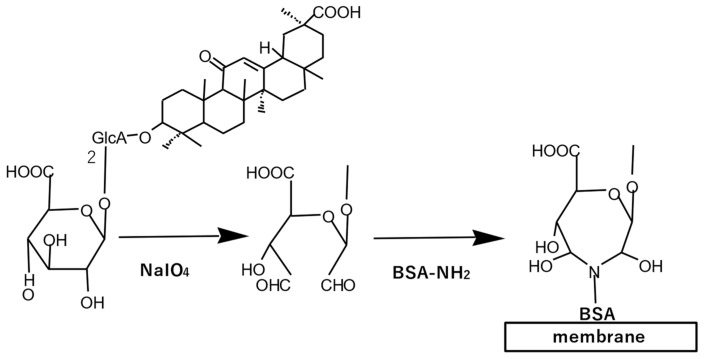
Eastern blotting scheme for glycyrrhizin. Glycyrrhizin blotted on a membrane after thin-layer chromatography was cleaved oxidatively to produce an intermediate form, which could be conjugated with bovine serum albumin resulting in its fixation onto the membrane, e.g., a polyvinylidene difluoride or a polyethersulfone membrane. Staining was performed using an anti-glycyrrhizin monoclonal antibody.

Southern, northern, and western blotting are accepted methodologies for the detection of large molecules, DNA/RNA, and proteins, respectively. As small-molecule natural products, glycosphingolipids and phospholipids were blotted onto a membrane from TLC plates using the so-called far-eastern blotting method, followed by detection [48]. We have developed a similar method called eastern blotting to detect glycosides in natural products. The staining procedure was discussed previously in relation to GC immunostaining in licorice root. The difference between far-eastern and eastern blotting is that eastern blotting contains a membrane conjugation step based on the oxidative cleavage of the glycoside, as indicated in Figure 5. This step promotes the attachment of small-molecule natural products to the membrane through covalent bonds. In other words, eastern blotting involves the separation of the above-described glycoside into an epitope (aglycone part) and a portion able to bind the membrane (sugar moiety). The first success of this technique was obtained with solasodine glycoside, in western blotting in 1997 [49]. The name of eastern blotting became established and unequivocal for GC in 2001 [15]. Subsequently, eastern blotting has been applied for natural products such as ginsenosides [39], GC [50], sennoside A and B [10], solasodine glycoside [49], and saikosaponin A [51].

**Figure 6:**
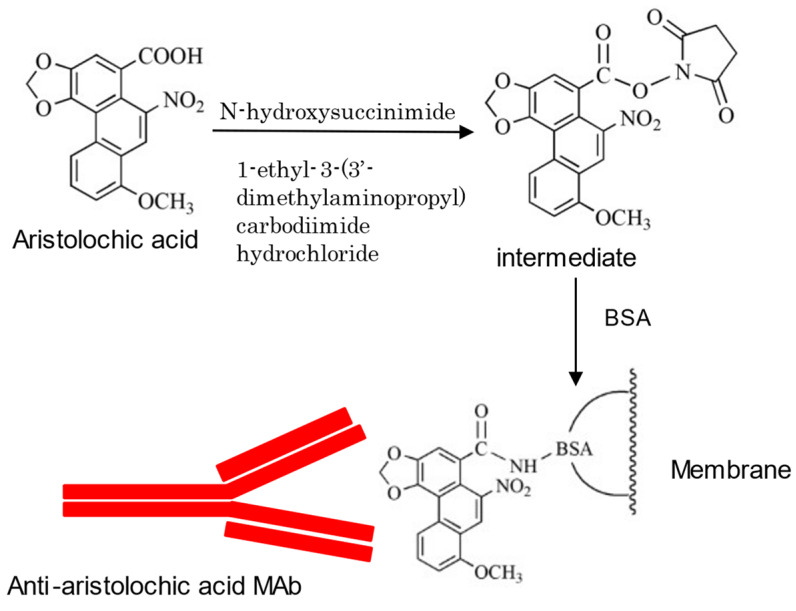
Eastern blotting approach for aristolochic acid (author’s unpublished data).

However, for non-glycoside compounds, such as AA, another method is used. AA was treated with N-hydroxysuccinimide and 1-ethyl-3-(3-dimethylaminopropyl) carbodiimide hydrochloride (EDC) to produce a semi-stable intermediate, which was then treated with BSA to yield an AA–BSA conjugate that could bind to a membrane, as indicated in Figure 6 [52].

**Figure 7:**
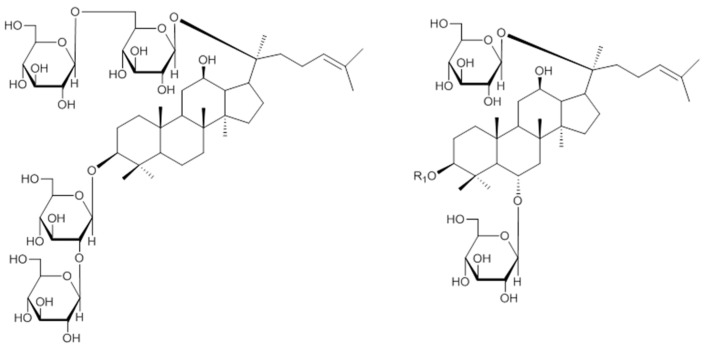
Ginsenoside Rb1 (**left**) and ginsenoside Rg1 (**right**) structures with protopanaxadiol and protopanaxatriol skeletons, respectively.

Previously, we named double eastern blotting the separate staining of two types of compounds by two sets of MAb and substrate. The double eastern blotting technology was successfully applied to ginsenosides using the anti-ginsenoside Rb1 and Rg1 MAbs [53]. In this case, protopanaxadiol- and protopanaxatriol-type ginsenosides (see Figure 7) could be distinguished easily by two MAb and substrate sets, and their further pharmacological activities could be recognized.

**Figure 8:**
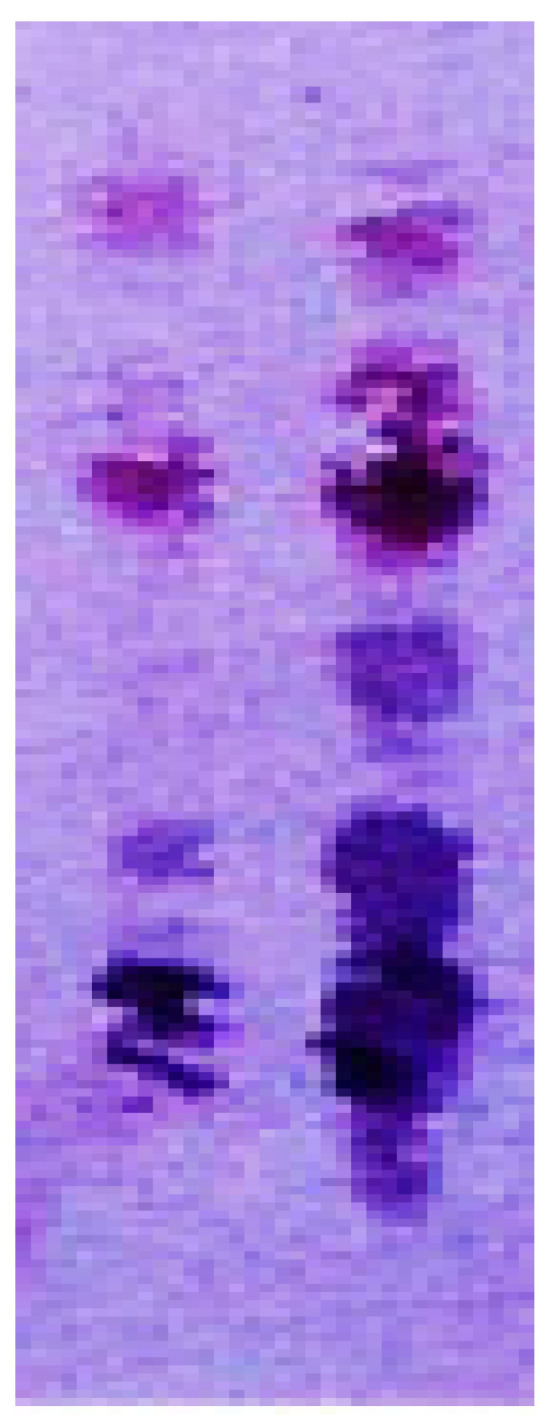
Double eastern blotting for ginsenosides contained in *Panax* species (author’s unpublished data) The thin-layer chromatography plate was developed with 1-butanol/water/acetic acid (7/2/1). The staining substrates were 4-chloro-1-naphthol (blue color for protopanaxadiol-type ginsenosides) and 3-amino-9-ethyrcarbazole (pinkish color for protopanaxatriol-type ginsenosides).

In Figure 8, the blue bands show protopanaxadiol-type ginsenosides, such as ginsenosides Rb1, Rc, Rd, quinqueoside-R1. The pinkish bands indicate protopanaxatriol-type ginsenosides, such as ginsenosides Re, Rf, Rg1, Rh1, notoginsenoside R1. The type of ginsenoside was indicated by the staining color, and the sugar number could be determined from the Rf value.

In order to determine the ginsenoside content in *P. japonicus*, its crude extract was separated using an immunoaffinity column containing an anti-ginsenoside Rb1 MAb. Since one spot indicated the same Rf value as that corresponding to ginsenoside Rb1 in eastern blotting, obtained by using an anti-ginsenoside Rb1 MAb, the sugar number was easily determined to be four. Based on this result, two ginsenosides, chikusetsusaponins III and IV, were identified as protopanaxadiol ginsenosides, although it was expected that *P. japonicus* might contain ginsenoside-Rb1 by enzyme-linked immunosorbent assay (ELISA) analysis [54].

Further double eastern blotting for compounds like sennoside A and B [55], GC, and liquiritin [56] was also carried out.

As a typical case, the eastern blotting of solasodine glycosides demonstrated that all solasodine glycosides could be stained by an anti-solamargine Mab, indicating a wide cross-reactivity for the majority of solasodine glycosides [49].

## 4. One-Step Purification on an Immunoaffinity Column

In general, the purification of peptides and/or proteins has been carried out by an immunoaffinity column conjugated with corresponding commercially available MAbs. On the other hand, for small-molecule natural products, hand-crafted affinity columns whose MAbs are prepared individually should be utilized because no commercial kits are available. Purified MAbs can be conjugated with an affinity gel to set up immunoaffinity columns. An immunoaffinity column is charged with the crude extract of natural products using a buffer; then an elution buffer containing an organic solvent is applied. The separated antigen compound fraction can be subjected to deionization and lyophilization to obtain a pure antigen. The authors succeeded in the one-step isolation of forskolin, a cyclic AMP activator, using an affinity column conjugated with an anti-forskolin Mab, following the above procedure, which provided 45 μg of pure forskolin from *Coleus forskolii* root (10 mg) [57]. Furthermore, ginsenoside Rb1 was also isolated from crude ginseng root by one-step immune affinity column containing an anti-ginsenoside Rb1 MAb [14] [58], although ginseng contains nearly 100 dammarane-type saponins [41]. MAbs on immune affinity columns are still active after being used 10 times. The amount of antigen purified by an immunoaffinity column is approximately 10 μg per mL of immunoaffinity gel; therefore laboratory-scale investigations can be performed. Immunoaffinity purification of natural additional products such as solasodine glycosides [59], glycyrrhizin [60], isoflavones [61], and naringin [30] has been performed.

## 5. Conclusions

MAb immunostaining is an essential technique in the field of life sciences, although it has not been applied in the field of small-molecule natural products so far. We developed a new staining method named eastern blotting, involving the separation of glycoside molecules into an epitope (aglycone moiety) and a portion with membrane-fixation ability (sugar component). The double eastern blotting system allows distinguishing aglycone differences such as those of protopanaxadiol and protopanaxatriol ginsenosides in ginseng. As discussed previously, double eastern blotting staining makes it possible to determine the structure of individual ginsenosides by the staining color, which depends on the aglycone, and by the Rf value linked to the sugar number. The set of MAb and substrate for ginsenosides with protopanaxadiol or protopanaxatriol as the aglycon can be selected so to obtain a coloring system with sufficient sensitivity for compound detection. Furthermore, the pharmacological activity of ginsenosides stained by double eastern blotting can be recognized because protopanaxadiol and protopanaxatriol ginsenosides have different activities, even though their structures are similar. This staining system is effective for the different components, as shown for licorice, such as a triterpene saponin, GC, and a flavonoid saponin, liquiritin, suggesting it can be used for a wide variety of natural products containing small molecules, through the separation of the glycoside function into an epitope (aglycone part) and a portion with membrane-fixation ability (sugar moiety). One-step isolation of small-molecule natural products by an immunoaffinity column containing a MAb is useful for the elucidation of these molecules’ function. During our investigation, we also found that the washing fraction contained all components except the target antigen compound and named it knockout extract. It is clear that the knockout extract could be used to elucidate the function of antigen components in crude extracts.

## Figures and Tables

**Table 1:** Monoclonal antibodies against natural products.

Component Plant	Plant Resource	Reference
Forskolin	*Coleus forskolii*	[17]
Crocin	*Crocus sativus*	[13]
Paeoniflorin, albiflorin	*Paeonia lactiflora*	[18]
Artemisinin, artesunate	*Artemisia annua*	[19]
Morphine, thebaine, codeine	*Papaver somuniferum*	[11]
Berbarine	*Coptis japonica*	[12]
Sennoside A, B	*Rhem spp.*, *Senna* spp.	[10]
Tetrahydrocannabinolic acid	*Cannavis sativa*	[20]
Gindgolic acid	*Ginkgo biloba*	[21]
Saikosaponin a	*Bupleurum falcatum*	[22]
Glycyrrhizin	*Glycyrrhiza* spp.	[15]
Ginsenoside Rb1, Rg1, Re	*Panax* spp.	[14,23,24]
Notoginsenoside R1	*Panax notoginseng*	[25]
Ginsenoside Rg3	*processed ginseng*	[26]
Ginsenoside Rh1	*Panax* spp.	[27]
Puerarin	*Pueraria lobata*	[28]
Paclitaxel	*Taxus brevifolia*	[29]
Naringin	*Citrus* spp.	[30]
Solamargine	*Solanum* spp.	[16]
Daidzin	*Glycine max*	[31]
Baicalin	*Scutellaria baicalensis*	[32]
Mitragynine	*Mitragyna speciosa*	[33]
Plumbagin	*Plumbago indica*	[34]
Aconitine	*Aconitum* spp.	[35]

## Data Availability

Data were taken from the references provided in the manuscript.
